# “Questionable” peer review in the publishing pandemic during the time of COVID-19: implications for policy makers and stakeholders

**DOI:** 10.3325/cmj.2020.61.300

**Published:** 2020-06

**Authors:** Francesco Chirico, Jaime A. Teixeira da Silva, Nicola Magnavita

**Affiliations:** 1 Post-Graduate Specialization of Occupational Health, Università Cattolica del Sacro Cuore, Rome, Italy *medlavchirico@gmail.com*; 2 Independent scholar, Kagawa-ken, Japan; 3 Università Cattolica del Sacro Cuore, Rome, Italy

To the Editor: We have read with interest the editorial by Škorić et al (1) in the April issue of the *Croatian Medical Journal*. The authors state that during the COVID-19 pandemic, the medical information system has been hit by a storm of open-access pandemic-related manuscripts through preprint platforms, but also through accelerated review processes.

Indeed, the urgency to tackle the COVID-19 pandemic and expand international collaboration has created the need to make scientific studies immediately available through PubMed Central and other sources such as the World Health Organization's Covid database (2). High-impact journals and publishers have established open-access platforms where researchers can publish reports of innovative responses to COVID-19, along with a range of opinion papers on policy and strategy relevant to the pandemic (3). Moreover, a group of publishers and scholarly communication organizations created a reviewer pool, supported by the Open Access Scholarly Publishers Association to “*maximize the efficiency of peer review, ensuring that key work related to COVID-19 is reviewed and published as quickly and openly as possible*” (4).

However, unprecedented scientific efforts have generated a torrent of publications, many of which are not peer-reviewed. It is even possible that indexed articles with a digital object identifier did not undergo peer review. A Reuters analysis of some of the most important servers (Google Scholar, *bioRxiv*, *medRxiv,* and *ChemRxiv*) indicated that 60% of studies were preprints, meaning that they reported non-peer-reviewed information (5).

This raises some concerns. First, non-peer-reviewed preprints could be cited by peer-reviewed articles published in legitimate peer-reviewed journals, possibly leading to the spread of misinformation. Second, peer review quality may be compromised by rushing the process and assigning an excessive workload to peer reviewers, thus putting them under pressure and inducing psychological stress. Third, the overabundance of opinion papers and editorials hinders the discovery of valuable raw data and medical insight. Finally, some data, ideas, and content, preliminary and peer-reviewed, are constantly being disproved, outdated, and invalidated, which makes them candidates for corrections or retractions following post-publication peer review. There is a real possibility that uncontrolled and potentially misleading information will reach the general public, directly or via the media, leading to incorrect, sometimes fatal, responses to the pandemic. In this scenario with many ethical challenges, scientific progress could be hampered, allowing predatory journals and scholars to exploit open access and possibly compromise public health and academic integrity.

Most of all, the spreading of misinformation “infodemic” through the social and traditional mass media poses a serious problem for public health systems. Restrictions imposed by policymakers and governments, such as lockdown and social distancing measures, are based on advice given by governments’ scientific advisory committees, which rely on scientific findings. However, the best scientific evidence available on COVID-19 is still scarce, making the decisions by governments susceptible to bias. Governments should not just base their decisions related to COVID-19 on scientific evidence (6), they should take full responsibility. Public health decision makers should consider the best scientific evidence available. For this reason, to cope with the explosion of information on COVID-19 it is particularly important to have high-quality systematic reviews (1). However, the methodological quality of most systematic reviews on previous coronavirus outbreaks is still poor, not only because of the speed in which they were completed (7), but also because of the apparently poor quality of peer review and editorial handling.
